# Sex Disparities in Diabetes Process of Care Measures and Self-Care in High-Risk Patients

**DOI:** 10.1155/2013/575814

**Published:** 2013-03-31

**Authors:** Margaret K. Yu, Courtney Rees Lyles, Luis A. Bent-Shaw, Bessie A. Young

**Affiliations:** ^1^Division of Nephrology, University of Washington, 1959 NE Pacific Street, Seattle, WA 98195, USA; ^2^Department of Epidemiology, University of Washington, 1959 NE Pacific Street, Seattle, WA 98195, USA; ^3^Kidney Research Institute, 325 9th Avenue, P. O. Box 359606, Seattle, WA 98104, USA; ^4^Department of Health Services, University of Washington, 1959 NE Pacific Street, Seattle, WA 98195, USA; ^5^Division of General Internal Medicine, UCSF, San Francisco, CA 94143, USA; ^6^Center for Vulnerable Populations, UCSF, 1001 Potrero Avenue, San Francisco, CA 94110, USA; ^7^Veterans Affairs Puget Sound Health Care System (152-E), Epidemiology Research and Information Center, 1660 S. Columbian Way, Seattle, WA 98108, USA

## Abstract

Patients with chronic diabetic complications experience high morbidity and mortality. Sex disparities in modifiable factors such as processes of care or self-care activities have not been explored in detail, particularly in these high-risk patients. Sex differences in processes of care and self-care activities were assessed in a cross-sectional analysis of the Pathways Study, an observational cohort of primary care diabetic patients from a managed care organization (*N* = 4,839). Compared to men, women had decreased odds of dyslipidemia screening (adjusted odds ratio (AOR) 0.73, 95% CI 0.62–0.85), reaching low-density lipoprotein goal (AOR 0.70, 95% CI 0.58–0.86), and statin use (AOR 0.69, 95% CI 0.58–0.81); women had 19% greater odds of reaching hemoglobin A1c <7% (95% CI 1.02–1.41). There were no sex differences in hemoglobin A1c testing, microalbuminuria screening, or angiotensin-converting enzyme inhibitor use. Women were less likely to report regular exercise but had better adherence to healthy diet, glucose monitoring, and self-foot examination compared to men. Patterns of sex differences were consistent in subjects with diabetic complications. Significant sex disparities exist in diabetes process of care measures and self-care, even amongst patients known to have chronic diabetic complications.

## 1. Introduction

In the United States, diabetes mellitus affects 26 million people [1] and its chronic vascular complications are associated with significant morbidity [2, 3], disability [4], and mortality [5]. Chronic complications from diabetes account for approximately $58 billion in excess medical expenditures per year [3]. Common risk factors for microvascular and macrovascular diabetic complications include age, duration of diabetes [6], hyperglycemia [7–9], and high blood pressure [10]; therefore multiple complications commonly develop in the same patient [5, 6]. In addition to the management of cardiovascular risk factors, prevention of diabetes complications also involves diabetes self-care such as diet, exercise, self-monitoring of blood glucose, and self-foot examination [11]. As a result, the American Diabetes Association (ADA) has established clinical practice guidelines regarding standard diabetes care, which include recommendations for diabetes process of care measures (frequency of laboratory testing, clinical goals, and recommended medications) and self-care [11].

A few studies have suggested that adherence with these diabetes clinical practice guidelines varies by sex. Women with diabetes have been reported to have worse blood pressure, lipid, and glycemic control compared to men [12], even amongst those known to have cardiovascular disease [13, 14]. Furthermore, diabetic women tend to be less physically active than men [15]. However, sex differences in laboratory testing and other self-care behaviors have not been explored in detail, nor is it known whether these sex disparities persist in high-risk patients, such as those who already have a history of a diabetic complication. Identification of modifiable factors related to diabetes outcomes is imperative if the rate of adverse outcomes is to be decreased, and evaluation of sex-specific differences provides an opportunity to develop strategies to reduce sex-related health disparities in diabetes care.

This study examined the associations between sex and selected diabetes process of care measures and self-care activities in a cohort of primary care patients with diabetes. This study also examined whether sex differences in diabetes process of care measures and self-care activities were detectable in the subgroup of subjects with a history of diabetic complications, a particularly high-risk group for adverse outcomes.

## 2. Materials and Methods

### 2.1. Participants

**Figure 1:**
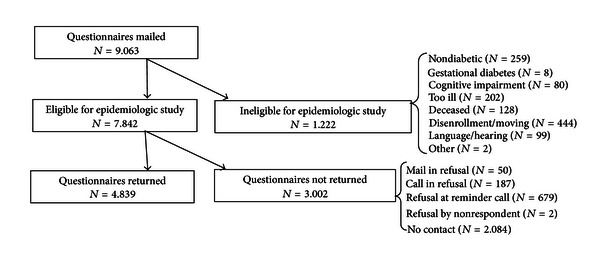
The Pathways Study subject recruitment.

We conducted a cross-sectional analysis of baseline data from the Pathways Study, which has been described previously [16, 17]. In brief, the Pathways Study is a prospective, observational cohort of the prevalence and impact of depression on patients with diabetes at Group Health (GH), a large nonprofit health maintenance organization (HMO) in Washington and Idaho, USA. GH maintains a registry of diabetes patients and their guideline-recommended test results. Nine primary care clinics were chosen for patient recruitment based on the number of diabetic patients, ethnic diversity, and proximity to Seattle, WA, USA. For the study, 9,063 potential candidates were identified from the GH diabetes registry (Figure 1). In 2001-2002, surveys were sent to these patients regarding demographic information, diabetes characteristics, diabetic complications, and self-care. Diabetic complications included retinopathy, nephropathy, neuropathy, cerebrovascular, cardiovascular, peripheral vascular disease, or metabolic (hypoglycemia, diabetic ketoacidosis, or hyperosmolar nonketotic coma). Of those identified, 1,222 patients were excluded from the study due to no diabetes, gestational diabetes, cognitive impairment, severe illness, deceased, disenrollment from GH, language or hearing problems, or other reasons. Of the remaining 7,842 eligible patients for the study, 4,839 (61.7%) returned the survey of which 4,467 (92.3%) gave permission to link survey data with automated data from GH regarding laboratory tests, pharmacy records, hospitalizations, and outpatient visits. The study protocol was approved by GH and University of Washington institutional review boards.

### 2.2. Measures

Baseline hemoglobin A1c and low-density lipoprotein (LDL) were ascertained closest to the date of the baseline epidemiologic survey, up to 12 months prior to study enrollment. Microalbuminuria was defined as a urine albumin to creatinine ratio (UACR) >17 mg/g for women and >25 mg/g for men, based on sex-specific cutoffs [18]; given the large proportion of missing data, microalbuminuria was ascertained up to 24 months prior to study enrollment.  Estimated glomerular filtration rate (eGFR) was calculated using Chronic Kidney Disease-Epidemiology (CKD-EPI) equations [19]. Chronic kidney disease (CKD) stage was determined by eGFR and microalbuminuria, using the National Kidney Foundation Kidney Disease Outcomes Quality Initiative classification system [20]. History of hypertension was based on ICD-9 code 401.x [21]. Computerized pharmacy records were used to identify patients who were prescribed any insulin, oral diabetic medication, HMG-CoA reductase inhibitor (statin), angiotensin-converting enzyme (ACE) inhibitor, or angiotensin receptor blocker (ARB) in the 12 months prior to study enrollment. For simplicity we will use the generic term ACE inhibitor to refer to either an ACE inhibitor or ARB.

Self-care activities were assessed using the Summary of Diabetes Self-Care Activities (SDSCA), which is a brief questionnaire that asks how many days per week an activity was performed [22]. The SDSCA has been shown to be a reliable and valid measure of adherence to diabetes self-care in observational and interventional studies [23]. For this study, investigators selected five SDSCA questions regarding diet, exercise, blood glucose testing, and foot care that were considered the most clinically relevant for analysis (Table 1). 

### 2.3. Outcomes

The primary outcomes of interest were sex-specific differences in the following diabetes process of care measures and self-care activities: (1) history of recommended laboratory testing (hemoglobin A1c, LDL, and microalbuminuria), (2) attainment of clinical targets (hemoglobin  A1c < 7% and LDL < 130 mg/dL), (3) medication use (statins in all subjects and in those with LDL >130 mg/dL, ACE inhibitors in all subjects and in those with microalbuminuria), and (4) compliance with self-care (diet, exercise, and foot examination at least 3 times per week; blood glucose testing at least 3 times a week if on oral hypoglycemic agents only or 5 times a week if on insulin). 

### 2.4. Data Analysis

Statistical analyses were performed using STATA version 12 (College Station, TX, USA) [24]. Significant sex differences in diabetes clinical process of care measures and self-care activities were determined using *t*-tests for continuous data and *χ* ^2^ tests for categorical data. Logistic regression models were used to calculate adjusted odds ratios (AORs) to determine if there were adjusted sex differences in compliance with diabetes process of care measures and self-care activities. Models regarding process of care measures were adjusted for age, race/ethnicity, marital status, education, smoking, body mass index, hemoglobin A1c (except for models where hemoglobin A1c testing and achievement of hemoglobin A1c <7% were outcomes of interest), history of hypertension, and CKD stage (except the model for microalbuminuria testing). Models regarding self-care activities were additionally adjusted for a history of major depression, which was strongly associated with both sex and self-care outcomes. Analyses were also performed on the subgroup of patients with a known history of at least one diabetic complication (*N* = 3,045), since these patients are at high risk for adverse outcomes. Sensitivity analyses demonstrated similar results in this subgroup as with subgroups of patients with specific diabetic complications (cardiovascular, cerebrovascular, or nephropathy).

## 3. Results

### 3.1. Pathways Cohort Characteristics

Of the 4,839 subjects in the total cohort, 48.8% were women (Table 2). Men tended to be older, more frequently married, and had higher levels of education and income compared to women. Women had higher mean BMI and greater prevalence of hypertension (45.0% versus 41.0%) and major depression (14.1% versus 9.6%) than men. Men had a greater prevalence of microalbuminuria (46.7% versus 34.4%) and higher mean number of diabetic complications (1.4 ± 1.4 versus 1.3 ± 1.3) compared to women. There were similar patterns of sex differences in cohort characteristics amongst the subset of subjects known to have diabetic complications.

### 3.2. Diabetes Process of Care Measures

Approximately 86.7% of subjects had previous hemoglobin A1c testing and 61.4% had urine microalbuminuria screening; these test frequencies did not vary significantly by sex. In contrast, LDL testing was less frequent in women compared to men; only 52.6% of women had their LDL checked in the previous year compared to 59.0% of men (*P* < 0.001).

Both men and women had a mean hemoglobin A1c of 7.8%, and a similar proportion of subjects achieved a hemoglobin A1c <7%. Mean LDL was higher in women (115.0 ± 36.0 mg/dL) than in men (107.8 ± 33.6 mg/dL, *P* < 0.001), and a lower proportion of women achieved a target LDL of <130 mg/dL (67.3% versus 75.3% in men, *P* < 0.001). Statins were prescribed less frequently to women than men overall (26.5% versus 35.4%, *P* < 0.001) and in those with LDL levels above 130 mg/dL (24.2% versus 31.3%, *P* = 0.02). There were no sex differences in ACE inhibitor use overall or in the subset with microalbuminuria.

In adjusted multivariable logistic regression models (Table 3), a greater proportion of women were more likely to be guideline discordant than men for LDL testing (AOR 0.73, 95% CI 0.62–0.85), achievement of LDL target <130 mg/dL (AOR 0.70, 95% CI 0.58–0.86), any statin prescription (AOR 0.69, 95% CI 0.58–0.81), or statin prescription if LDL was greater than 130 mg/dL (AOR 0.61, 95% CI 0.41–0.91) compared to men. Women were more likely to achieve hemoglobin A1c target (AOR 1.19, 95% CI 1.02–1.41). There were no differences in the odds of hemoglobin A1c testing, microalbuminuria screening, or ACE inhibitor prescription by sex.

### 3.3. Diabetes Self-Care Activities

Women reported more frequent consumption of ≥5 servings of fruits or vegetables per day and less frequent consumption of high fat foods compared with men. In the week prior to study assessment, a lower proportion of women (55.3%) exercised at least 3 times a week compared to men (63.9%, *P* < 0.001). Frequency of blood glucose testing was similar by sex. Women tended to examine their feet more frequently than men.

**Figure 2:**
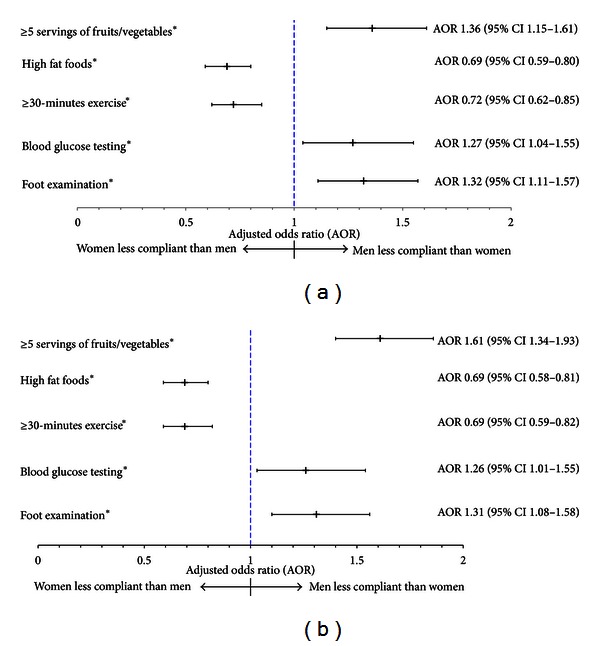
Logistic regression models of compliance with self-care activities for women compared to men in the overall cohort (a) and amongst subjects with at least one diabetic complication (b). Adjusted for age, race/ethnicity, marital status, education, smoking, body mass index, hemoglobin A1c (except model for hemoglobin A1c <7%), history of hypertension, and major depression. Models in (a) were also adjusted for chronic kidney disease stage.

In adjusted logistic regression models of self-care activities (Figure 2(a)), women were more likely to report high fruit and vegetable consumption (AOR 1.36, 95% CI 1.15–1.61), blood glucose testing (1.27, 95% CI 1.04–1.55), and self-foot examination (AOR 1.32, 95% CI 1.11–1.57) but less likely to report fatty food consumption (AOR 0.69, 95% CI 0.59–0.80) and regular exercise (AOR 0.72, 95% CI 0.62–0.85) compared to men.

### 3.4. Patients with Diabetic Complications

Based on adjusted logistic regression models of 3,045 patients with at least one known diabetic complication, men and women had similar odds of hemoglobin A1c and microalbuminuria testing (Table 4). Women in this subgroup had decreased odds of LDL testing (AOR 0.65, 95% CI 0.54–0.77), achievement of LDL goal <130 mg/dL (AOR 0.63, 95% CI 0.51–0.78), statin use overall (AOR 0.67, 95% CI 0.56–0.79), and statin use if serum LDL was >130 mg/dL (AOR 0.64, 95% CI 0.42–0.97). Women with at least one diabetic complication had greater odds of achieving target hemoglobin A1c <7% compared to their male counterparts. There were no sex-specific differences in ACE inhibitor use.

Amongst those with at least one diabetic complication, women had greater odds of high fruit and vegetable consumption (AOR 1.61, 95% CI 1.34–1.93), blood glucose monitoring (AOR 1.26, 95% CI 1.01–1.55), and self-foot examination (AOR 1.31, 95% CI 1.08–1.58) compared to men (Figure 2(b)). Women in this subgroup were less likely to consume fatty foods (AOR 0.69, 95% CI 0.58–0.81) or to exercise (AOR 0.69, 95% CI 0.59–0.82) compared to their male counterparts.

## 4. Discussion

This analysis found significant sex differences in diabetes process of care measures and self-care activities, even amongst the high-risk subgroup of subjects who were known to have chronic complications of diabetes. In general, women tended to have better glycemic control and adherence to recommended self-care compared to men. However, despite having higher LDL levels, women were less likely to be screened for dyslipidemia or to be prescribed statins compared to men. Women were also less likely to engage in physical activity than men.

This is the first study to report sex disparities in diabetes processes of care and self-care behaviors in the subgroup of patients with a history of at least one diabetic complication, which is surprising since these patients are at high-risk for additional diabetic complications and warrant aggressive diabetes care. Our results are congruent with previous findings of sex differences in diabetes process of care measures, particularly with respect to management of dyslipidemia. In a cross-sectional study of 3,849 patients with diabetes from five academic medical centers, Wexler et al. found that women had higher cholesterol levels, were less likely to receive lipid lowering therapy, and when receiving lipid lowering therapy, were less likely to reach LDL targets compared to men [14]. Ferrara et al. found that, compared to men, diabetic women without cardiovascular disease received less frequent lipid testing and diabetic women with cardiovascular disease were treated less frequently with lipid lowering agents [25]. Although other studies have reported worse glycemic control in diabetic women compared to men [12, 14], we found that women had better glycemic control than men. 

We also found significant sex differences in patterns of diabetes self-care. Diabetic women tended to be more physically inactive than men, which is consistent with findings from the Third National Health and Nutrition Examination Survey (NHANES III) [15]. This study builds on previous reports by demonstrating sex differences in other diabetes self-care activities. Diabetic men were consistently less adherent to recommendations regarding diet, blood glucose monitoring, and foot care than women. Likewise, these sex differences in diabetes self-care persisted in patients with known diabetic complications.

The observed sex differences in diabetes process of care measures and self-care activities are likely multifactorial and may be related to both provider and patient factors. Providers may perceive diabetic women to have a lower risk of cardiovascular disease and other diabetic complications compared to men, which may result in less aggressive monitoring and treatment in women. Whether sex influences management of diabetic patients has not been investigated; however, research in cardiovascular disease revealed that sex influenced how physicians managed chest pain [26], coronary heart disease [27], and cardiovascular disease prevention [28]. Since public awareness of cardiovascular disease in women remains suboptimal [29, 30], female patients may themselves underestimate their risk for diabetic complications and either fail to inquire about or decline routine diabetes care. Moreover, men and women may have differing beliefs in the benefit of self-care. In a survey of new patients to a diabetes education center, women were more likely than men to have a history of previous diabetes education and had higher expectations that self-management would improve health outcomes; this may explain our finding that women had better glycemic control and overall better adherence to self-care than men [31]. Finally, biologic differences between men and women, due to estrogen or lack thereof, may result in differential outcomes in care. Sex hormones are associated with glucose tolerance [32], lipid metabolism [33], albuminuria [34], and coronary heart disease [35]. Diabetes has a greater adverse effect on serum triglyceride and LDL levels in women compared to men [36], and a recent meta-analysis of statin therapy for secondary cardiovascular prevention found that statins reduced all-cause mortality and stroke risk men but not in women [37]. 

It is important to recognize that the results of this study present an opportunity to improve the quality of diabetes care not only for women, but for men as well. Although women had less adequate screening and management of dyslipidemia and poorer adherence to exercise, they tended to have better glycemic control and adherence to other recommended self-care activities compared to men. Health care providers should pay particular attention to ordering recommended laboratory tests and medications in women and targeting patient education interventions regarding self-care to men. All patients should be encouraged to follow a regular exercise program and this should be heavily emphasized among women.

The strengths of this study include the large sample size of study subjects with comparable access to care. The study surveyed patients directly for variables related to diabetes self-care and we were able to adjust for several known confounding variables, including major depression. However, this study does have several limitations that are important to consider. The cross-sectional nature of this study is subject to unmeasured confounding and cannot establish causal relationships. We did not have access to actual blood pressure measurements and therefore could not adjust for the degree of control of hypertension. There was a large proportion of missing data for laboratory test results, particularly for LDL and microalbuminuria, which affects the validity and generalizability of these results. Pharmacy records could only capture medications prescribed within the GH system and do not reflect actual patient usage. Self-care activities were ascertained by self-reported measures rather than actual measurements. Although the SDSCA has been shown to be a reliable and valid measure of diabetes self-management [22], differential misclassification could occur if there were systematic differences in how men and women recall or report self-care. Finally, this study could not account for several factors that may contribute to the observed sex differences, such as differences in patient visit frequency, provider styles of care, or patient preferences by sex.

## 5. Conclusion

In conclusion, sex is associated with significant differences in diabetes process of care measures and self-care activities, even amongst subjects known to have chronic complications from diabetes. Women may benefit from more attention to dyslipidemia screening, lipid lowering treatment, and regular exercise, whereas men may require more encouragement in diabetes self-care including healthy diet, self-blood glucose monitoring, and self-foot examination. The findings from this study indicate an opportunity for intervention to reduce sex-related disparities in diabetes care. Although further studies are needed to elucidate the causes for these sex disparities, it is important for primary health care providers to be aware of the existence of sex differences in diabetes care such that these disparities may be eliminated.

## Figures and Tables

**Table 1:** Selected questions from the Summary of Diabetes Self-Care Activities (SDSCA).

Self-care domain	Description	SDSCA question
Diet	≥5 servings of fruits/vegetables	On how many of the last SEVEN DAYS did you eat five or more servings of fruits and vegetables?
Diet	High fat foods	On how many of the last SEVEN DAYS did you eat high fat foods such as red meat or whole-fat dairy products?
Exercise	≥30 minutes exercise	On how many of the last SEVEN DAYS did you participate in at least 30 minutes of physical activity? (This means 30 minutes of continuous activity, including walking)
Blood glucose	Blood glucose testing	On how many of the last SEVEN DAYS did you test your blood sugar?
Foot care	Foot examination	On how many of the last SEVEN DAYS did you check your feet?

**Table 2:** Pathways cohort characteristics by sex.

	All subjects			Subjects with diabetic complications		
	Women (*N* = 2,360)	Men (*N* = 2,479)	*P*	Women (*N* = 1,445)	Men (*N* = 1,600)	*P*
Age (years)	62.5 ± 14.0	63.8 ± 12.7	0.002	64.4 ± 13.6	66.0 ± 12.1	0.001
Race/ethnicity						
Non-Hispanic white	1,753 (74.3)	1,872 (75.5)	0.62	1,104 (76.4)	1,245 (77.8)	0.59
Non-Hispanic black	207 (8.8)	195 (7.9)		132 (9.1)	124 (7.8)	
Asian/Pacific Islander	236 (10.0)	236 (9.5)		119 (8.2)	131 (8.2)	
Other	164 (7.0)	176 (7.1)		90 (6.2)	100 (6.3)	
Married	1,238 (53.3)	1,924 (78.4)	<0.001	742 (52.1)	1,252 (78.7)	<0.001
≥High school education	1,640 (71.0)	1,918 (78.6)	<0.001	987 (69.7)	1,234 (77.9)	<0.001
Income ≥ $20,000/year	931 (51.4)	1,264 (61.9)	<0.001	521 (47.7)	755 (57.3)	<0.001
Smoker	181 (7.9)	231 (9.4)	0.05	102 (7.2)	125 (7.9)	0.47
Body mass index (kg/m^2^)	32.3 ± 8.2	29.7 ± 5.7	<0.001	32.3 ± 8.2	29.8 ± 5.8	<0.001
Hypertension	980 (45.0)	939 (41.0)	0.008	753 (52.1)	758 (47.4)	<0.001
Major depression	331 (14.1)	237 (9.6)	<0.001	211 (14.7)	186 (11.6)	0.01
Diabetic complications						
Number of diabetic complications	1.3 ± 1.3	1.4 ± 1.4	<0.001	1.9 ± 1.1	2.0 ± 1.1	0.006
≥1 diabetic complication	1,445 (66.3)	1,600 (69.9)	0.01	—	—	—
Microalbuminuria	507 (34.4)	698 (46.7)	<0.001	486 (50.5)	633 (61.3)	<0.001
Laboratory testing						
Hemoglobin A1c (12 months)	2,043 (86.6)	2,153 (86.9)	0.77	1,366 (94.5)	1,521 (95.1)	0.51
LDL (12 months)	1,242 (52.6)	1,462 (59.0)	<0.001	843 (58.3)	1,082 (67.6)	<0.001
Microalbuminuria (24 months)	1,475 (62.5)	1,494 (60.3)	0.11	963 (66.6)	1,032 (64.5)	0.21
Laboratory results						
Mean hemoglobin A1c (%)	7.8 ± 1.5	7.8 ± 1.6	0.41	7.9 ± 1.6	7.9 ± 1.6	0.64
Hemoglobin A1c < 7%	692 (33.9)	683 (31.7)	0.14	417 (30.5)	429 (28.2)	0.17
Mean LDL (mg/dL)	115.9 ± 36.0	107.8 ± 33.6	<0.001	112.3 ± 35.3	104.9 ± 33.3	<0.001
LDL < 130 mg/dL	955 (67.3)	1,259 (75.3)	<0.001	671 (70.3)	969 (78.5)	<0.001
Medications						
Statin	577 (26.5)	809 (35.4)	<0.001	451 (31.2)	665 (41.6)	<0.001
Statin if LDL >130 mg/dL	110 (24.2)	122 (31.3)	0.02	81 (29.5)	93 (36.9)	0.07
ACE inhibitor	1,238 (56.8)	1,354 (59.2)	0.11	901 (62.4)	1,040 (65.5)	0.07
ACE inhibitor if microalbuminuria	342 (67.5)	455 (65.2)	0.41	334 (68.7)	424 (67.0)	0.54
Diet						
≥5 servings of fruits/vegetables (days/week)	4.1 ± 2.4	3.8 ± 2.5	<0.001	4.1 ± 2.4	3.8 ± 2.5	0.004
High fat foods (days/week)	2.8 ± 1.9	3.2 ± 2.0	<0.001	2.8 ± 1.9	3.1 ± 2.0	<0.001
Exercise						
≥30-minute exercise (days/week)	3.0 ± 2.4	3.5 ± 2.4	<0.001	2.8 ± 2.4	3.4 ± 2.4	<0.001
≥30-minute exercise, 3 times a week	1,276 (55.3)	1,568 (63.9)	<0.001	730 (51.8)	989 (62.4)	<0.001
Blood glucose testing (days/week)	4.5 ± 2.8	4.4 ± 2.9	0.23	4.7 ± 2.8	4.6 ± 2.8	0.34
Foot examination (days/week)	4.7 ± 2.6	4.4 ± 2.8	<0.001	4.8 ± 2.6	4.7 ± 2.7	0.09

Data are *n* (%) or mean ± SD.

LDL: low-density lipoprotein; ACE: angiotensin converting enzyme.

**Table 3:** Logistic regression models of diabetes process measures for women compared to men in the pathways study.

	Unadjusted OR (95% CI)	*P*	Adjusted OR (95% CI)*	*P*
Hemoglobin A1c testing (12 months)	0.98 (0.83–1.15)	0.77	1.04 (0.68–1.61)	0.85
LDL testing (12 months)	0.77 (0.69–0.87)	<0.001	0.73 (0.62–0.85)	<0.001
Microalbuminuria testing (24 months)	1.10 (0.98–1.23)	0.11	1.15 (0.99–1.34)	0.07
Hemoglobin A1c < 7%	1.10 (0.97–1.25)	0.14	1.19 (1.02–1.41)	0.03
LDL < 130 mg/dL	0.68 (0.58–0.79)	<0.001	0.70 (0.58–0.86)	0.001
Statin	0.66 (0.58–0.75)	<0.001	0.69 (0.58–0.81)	<0.001
Statin if LDL >130 mg/dL	0.70 (0.52–0.95)	0.02	0.61 (0.41–0.91)	0.02
ACE inhibitor	0.91 (0.81–1.02)	0.11	0.97 (0.83–1.14)	0.71
ACE inhibitor if microalbuminuria	1.11 (0.87–1.41)	0.41	1.14 (0.86–1.50)	0.36

*Adjusted for age, race, marital status, education, smoking, body mass index, hemoglobin A1c (except models for hemoglobin A1c testing and hemoglobin A1c <7%), history of hypertension, and chronic kidney disease stage (except model for microalbuminuria testing).

LDL: low-density lipoprotein; ACE: angiotensin converting enzyme.

**Table 4:** Logistic regression models of diabetes process measures for women compared to men amongst subjects with diabetic complications.

	Unadjusted OR (95% CI)	*P*	Adjusted OR (95% CI)*	*P*
Hemoglobin A1c testing (12 months)	0.90 (0.65–1.23)	0.51	0.94 (0.66–1.34)	0.75
LDL testing (12 months)	0.67 (0.58–0.78)	<0.001	0.65 (0.54–0.77)	<0.001
Microalbuminuria testing (24 months)	1.10 (0.95–1.28)	0.21	1.06 (0.88–1.28)	0.51
Hemoglobin A1c < 7%	1.12 (0.95–1.31)	0.17	1.21 (1.01–1.44)	0.04
LDL < 130 mg/dL	0.65 (0.54–0.79)	<0.001	0.63 (0.51–0.78)	<0.001
Statin	0.64 (0.55–0.74)	<0.001	0.67 (0.56–0.79)	<0.001
Statin if LDL >130 mg/dL	0.71 (0.50–1.03)	0.07	0.64 (0.42–0.97)	0.04
ACE inhibitor	0.87 (0.75–1.01)	0.07	0.87 (0.73–1.03)	0.10
ACE inhibitor if microalbuminuria	1.08 (0.84–1.40)	0.54	1.10 (0.83–1.47)	0.51

*Adjusted for age, race, marital status, education, smoking, body mass index, hemoglobin A1c (except models for hemoglobin A1c testing and hemoglobin A1c <7%), and history of hypertension.

LDL: low-density lipoprotein; ACE: angiotensin converting enzyme.
